# Poisoning by Sir William Burnett’s Disinfecting Fluid

**Published:** 1853-11

**Authors:** Richard Hassall


*Poisoning by Sir William Burnett's Disinfecting Fluid.* By Richard Hassall, M. D.—The first case I have to relate is one of unusual interest. In the first place, the instances of poisoning by chloride of zinc which have been placed on record are very few; in the second place, the train of nervous symptoms observed was of a most remarkable character; in the third, the efficacy of prompt treatment is illustrated in a very striking manner.

In the systematic writing of our leading toxicologists no mention is made of chloride of zinc as a poisonous substance; the only salt of zinc referred to as possessing any interest in this respect is the sulphate. Even Dr. Taylor, in his elaborate work on "Poisons," bestows upon the chloride but the most cursory notice, and the subject is evidently suggested to him rather by the methodical arrangement of his matter than from any precise information as to its toxicological properties.

Dr. Stretton has recorded two cases of poisoning by chloride of zinc. In one, twelve grains of the salt were taken; in the other, two hundred grains dissolved in a wineglass full of water. Both the patients recovered. In the *Medico-Chirurgical Transactions* for 1850, a most interesting case, which terminated fatally, is detailed by Dr. Letheby; and it is to the same paper that we must look for whatever accurate knowledge we possess of the poisonous properties of this substance.

In May, 1851, a man who a week previously had taken some of Burnett's fluid by mistake for gin became a patient at the Metropolitan Free Hospital. His first symptoms were described to have been severe pains in the stomach, distressing vomiting, frothing at the mouth, and a livid countenance. He appears to have got over the immediate effects of the poison, without any treatment whatever. After a week there was great emaciation, weakness, pain in the stomach, and constant sickness. Under treatment, which does not seem to have been guided by any precise indications, he gradually recovered. A few other cases are casually reported, but too concisely to permit of any definite conclusions.

That instances of poisoning by chloride of zinc should be rare is a matter of surprise, when we consider how common is the use of Sir William Burnett's fluid; and when attention is called to the circumstance which led to the accident I am about to relate, this surprise will be very much increased. A gentleman was very much in the habit of having recourse to a draught of Murray's fluid magnesia for the relief of occasional sickness. One day, being about to sit down to dinner, sickness supervening, he called for his usual draught. About three ounces by measure of a liquid was brought to him, and rapidly swallowed. It turned out that a bottle of Sir William Burnett's fluid was in the house for disinfecting purposes; out of this bottle my patient had been supplied. On comparing the two bottles, it was observed that their size and shape were closely similar—the resemblance even extended to the labels; and in short the general aspect of the two was so nearly identical as readily to favor a mistake. Sir James Murray's fluid magnesia has been in general repute for many years; and it is very much to be regretted that in introducing Sir W. Burnett's fluid care was not taken to adopt some distinctive character for the bottles. The risk of mistaking a dangerous poison for a harmless medicament ought surely to be avoided.

On the 6th of November, 1852, at about six P. M., three ounces by measure—probably four ounces three drachms, troy, by weight—of Sir Wm. Burnett's disinfecting fluid were hastily swallowed, as I have already mentioned. As this took place just before dinner, it may be inferred that the stomach was empty. This circumstance it is important not to pass over, since it must have operated in a two-fold manner to augment the danger of the case. The irritant and even corrosive fluid was brought into immediate contact with the coats of the stomach, and the system would be in the most favorable condition for absorption.

The first symptom experienced was a sense of constriction in the fauces, and then a hot, burning sensation in the stomach marked the arrival of the fluid in that organ. There was no pain in the mouth, or even in the œsophagus, nor was there any appearance of corrosion about the lips or mouth. This I account for by the suddenness with which the draught was gulphed down, leaving no time for any local effect. Almost immediately after the accident, the case was also seen by Mr. Hancock, Dr. Seaton, and my brother, Dr. Arthur Hassall. Within two minutes a quantity of water was swallowed, and vomiting was excited by thrusting the finger down the fauces. Ten minutes were consumed in getting ready a quantity of eggs and milk; when these were procured they were liberally administered. Vomiting was renewed by irritating the fauces, but it is remarkable that it did not set in spontaneously for nearly an hour. After this time the mixture was thrown off instantly after being taken, by the most violent action; it returned as a solid, coagulated mass; one portion of which was so large and so firm as almost to cause suffocation. This treatment was kept up throughout the night, and until two P. M. of the following day, the patient vomiting continuously, and rejecting the milk and eggs in the same curdled state. In the evening blood appeared in the vomited matter, a quantity of mucus, and a portion of mucous membrane measuring an inch and a quarter square. Throughout the third day the vomited matter was still streaked with blood. There was no action of the bowels until the third day, when they acted spontaneously; the stool was of a black, coffee-ground appearance, and entirely wanting the odor of fæces. The urine presented nothing unusual.

From the beginning the patient experienced the most distressing headache, and utter inability to sleep, and violent "clawing" pain in the stomach. This "clawing" in the stomach persisted in great severity for a long time, and even now (March, 1853) is felt when the stomach is empty. On this (the third) day, the pain described was felt intensely, but there was no tenderness on pressure; the pulse was 90, and had no remarkable character. Leeches were applied to the epigastrium, and followed by poultices. This gave no relief; indeed this was only obtained by taking bland, warm things, such as gruel, arrow root, and milk. Opium brought no ease. There was an excessive nervous derangement and prostration, tremor of the limbs, and a great disposition to pick the bedclothes; a feeling of sinking, and an urgent craving for food; the tongue was intensely red.

It was quite a fortnight before anything but black stools appeared; then they were pale, clayey, dry, and crumbling, showing no evidence of bile. The bowels being, moreover, sluggish, an occasional pill of blue-pill and rhubarb, and a draught of senna and tartrate of soda, were taken. These gave no pain or inconvenience; the stools gradually improved, but it was six weeks before the proper proportion of bile appeared, and they assumed a natural appearance. Throughout this time there was no natural appetite, but a morbid craving for something wherewith to allay the irritation of the stomach. From the third day the skin assumed a ghastly bluish-green tint; this lasted for a long time, and has not even yet quite disappeared. Great emaciation was observed; the skin looked as if it was stretched tightly over the bones of the face and hands.

I have now to detail the curious train of nervous symptoms which attended this case. The prostration, muscular tremors, wakefulness, were early observed. The sense of smell underwent a singular perversion: every kind of roast meat, if ever so slightly burnt, roasted coffee, tea, &c., had to my patient an intolerable putrid odor; roast mutton was especially offensive. Scents and perfumes prepared with alcohol gave an odor resembling that of hemlock; on the other hand, putrid and fæcal matter had no apparent odor at all.

The perversion of taste was not less remarkable; and the points of resemblance between the phenomena of taste and smell, served to illustrate the relation existing between these senses. Roast meats and crusts of bread were intolerable; and most foods had a burnt taste, especially *raw oysters*, which resembled burnt flour, and felt quite dry, clinging to the mouth. Things naturally insipid, such as the crumb of bread and potato, gave a more natural flavor and were better relished. Acids did not taste acid, nor alkalies alkaline, and to him quinine was not bitter.

These peculiarities continued strongly marked for some time, and the senses of smell and taste are still imperfect.

I have now to return to the history of the case. A gradual improvement was made for the first two months, the urgency of the symptoms described having subsided. Too early a return to accustomed avocations, while the strength was still low and the nervous system had not yet recovered its equilibrium, brought on an attack of illness of extreme severity. The patient was seized with symptoms resembling the onset of influenza; there was excessive nervous prostration, hyperæsthesia, and great irritability, which passed into a condition resembling tetanus; no noise could be borne; the slightest shock caused a start; if the bedclothes touched the toes, tremors approaching convulsions ensued. For five nights there was no sleep. There was a terrible pain in the right side of the head, which would last for hours; when this subsided, nausea and retching ensued; the pulse rose to 120 or 130. There was some febrile movement; the skin was alternately very dry and moist. During this secondary attack the liver again evinced a sluggishness of action, and the urine, which hitherto had been normal in quantity, became excessive; on one occasion seven pints were passed in five hours. In the first instance blue-pill and ipecacuanha and salines were ordered. For fourteen days the patient was confined to his bed, when the intensity of the symptoms had subsided, he still complained of the gnawing of the stomach. Dr. Wilson was consulted, and magnesia, with bismuth and hydrocyanic acid, were prescribed, and attended with some relief. A gradual improvement has again taken place, but the natural hue of the skin has not quite returned; the taste and smell are still impaired; the pain in the stomach is occasionally felt, and the general strength is reduced.

I will not lengthen this communication by entering upon a minute analysis of the symptoms observed, but I must beg to arrest attention to some points of especial interest. The poisonous action of chloride of zinc—that is, in that state of concentration in which it is found in Burnett's fluid—is of a double character: it has a local corrosive action, and a general toxical effect, the result of absorption.

I have already endeavored to account for the absence of any appearance of corrosive action in the present case about the lips or mouth by the rapidity of swallowing. In Dr. Letheby's case, however, corrosion was marked; "the lips were swollen; their inner surface and the lining membrane of the mouth presented an opaque white appearance;" and "when the child attempted to swallow water the greater part returned by the nostrils." The local action of the poison was also marked by the "hard and leathery" consistence of the stomach after death; but although in the case before us the mouth and œsophagus escaped, I cannot doubt but the stomach suffered; the vomiting of blood, the shred of mucous membrane rejected, and the passage of blood by stool indicate some sign of local injury; and this was the more likely to occur from the stomach being empty. Had food been present the chemical action of the poison would have been spent upon it, and the coats of the stomach would have been more or less protected. One circumstance in connexion with this local action deserves especial notice on account of its bearing on the treatment of similar cases of poisoning. It might, *prima facie*, be concluded, that the salt would speedily be decomposed, and removed in altered combinations by a moderate supply of albuminous matter and vomiting; but this does not appear to be the case. An enormous quantity of eggs and milk was passed through the stomach, and yet for many hours it was constantly rejected, strongly coagulated from the action of the poison. Dr. Letheby also detected a considerable quantity of chloride of zinc in the tissues of the stomach in his case, although vomiting had taken place. From this consideration, then, fortified by the successful issue of the case, I would strongly urge the necessity not only of a copious but a long-continued supply of albuminous matters. I think it the more important to refer to this point, because the treatment of poisoning by chloride of zinc is still unsettled or misunderstood. In one of Dr. Stretton's cases, soap-suds were administered, and in the case referred to at the Metropolitan Free Hospital. The physician who had charge of it, in reporting the history, recommended that soap or carbonate of potash or soda should be given in order to throw down carbonate of zinc. This practice is doubtful. The carbonate of zinc, although insoluble, might still be injurious; certainly calomel, carbonate of lead, sulphate of baryta, and other substances, cannot be said to be inert. The constitutional effects of the poison in this case are well marked; one of the earliest manifestations of the absorption of the poison into the blood is remarkable. The ghastly, bluish-green color of the skin can be attributed to no other cause than to the action of the poisonous compound (probably more especially of the chlorine) on the blood. It is unnecessary to refer at length to the nervous symptoms already detailed. It must be difficult to analyze the constitutional symptoms, and to determine how much is to be attributed to the hydrochloric acid and how much to the zinc. The experiments, however, of Orfila, Blake and Letheby, seem to prove that the nervous symptoms are dependent upon the zinc. Dr. Letheby is of opinion that the poison enacts a distinct and specific action on the motor and organic system of nerves. The excessive prostration, the strange perversion of the senses of smell and taste, the tremors of the limbs, and the tendency to tetanoid convulsions, observed by myself, would appear to corroborate this opinion.

As the recorded cases of poisoning by chloride of zinc show that this substance does not generally cause death in consequence of its primary corrosive action, it is a point of especial interest to consider by what means the elimination of the salt from the system can be promoted, and its secondary effects obviated or mitigated. The three great depurating sources are the liver, the kidney, and the skin; but it requires some caution before determining upon stimulating these organs to action. As in the case of poisoning by arsenic, chloride of zinc appears strongly to irritate, if not to inflame, the kidneys. In Dr. Letheby's case, the kidneys were greatly congested; in my case, the kidneys did not appear in the first instance to be much affected. The effect upon the liver was more marked; the arrest of its action was no doubt one reason why the poison was so long delayed in the system. Possibly more advantage might have been taken of the favorable condition of the kidneys. Are we to regard the excessive diuresis which marked the second attack as an effort of Nature to get rid of the poison remaining in the system? The peculiar character of the symptoms attending that attack clearly show that the system was still laboring under its effects. Before putting an increased strain upon kidneys, however, we must take care to ascertain that they are free from any congestion or inflammation. If they should be in this condition, then we must turn to the liver, and especially the intestines. Saline purgatives, with jalap, might be employed with advantage; and at the same time every effort should be made to promote the free action of the skin; warm baths and friction would be of most material assistance. M. Bonvier, in treating cases of zinc-poisoning among workmen engaged in oxide of zinc manufactories, detected zinc in the washings of the skin. A similar fact has been long known with reference to lead-poisoning.

I cannot quit this case without once more insisting upon the propriety of so altering the appearance of the bottles in which Burnett's Fluid is sold as to obviate the possibility of its being again taken by mistake for fluid magnesia.—*Lancet.*

